# Comparing two methods for deriving dietary patterns associated with risk of metabolic syndrome among middle-aged and elderly Taiwanese adults with impaired kidney function

**DOI:** 10.1186/s12874-020-01142-4

**Published:** 2020-10-14

**Authors:** Adi Lukas Kurniawan, Chien-Yeh Hsu, Hsiu-An Lee, Hsiao-Hsien Rau, Rathi Paramastri, Ahmad Syauqy, Jane C.-J. Chao

**Affiliations:** 1 School of Nutrition and Health Sciences, College of Nutrition, Taipei Medical University, 250 Wu-Hsing Street, Taipei, 11031 Taiwan; 2 Research Center for Healthcare Industry Innovation, National Taipei University of Nursing and Health Sciences, 365 Ming-Te Road, Peitou District, Taipei, 11219 Taiwan; 3 Department of Information Management, National Taipei University of Nursing and Health Sciences, 365 Ming-Te Road, Peitou District, Taipei, 11219 Taiwan; 4 Master Program in Global Health and Development, College of Public Health, Taipei Medical University, 250 Wu-Hsing Street, Taipei, 11031 Taiwan; 5 Department of Computer Science and Information Engineering, Tamkang University, 151 Yingzhuan Road, Tamsui District, New Taipei, 25137 Taiwan; 6 Joint Commission of Taiwan, 31 Sec.2 Sanmin Road, Banqiao District 220, New Taipei City, Taiwan; 7 Department of Nutrition Science, Faculty of Medicine, Diponegoro University, Jl. Prof. H. Soedarto, SH, Tembalang, Semarang City, Central Java 50275 Indonesia; 8 Nutrition Research Center, Taipei Medical University Hospital, 252 Wu-Hsing Street, Taipei, 11031 Taiwan

**Keywords:** Dietary pattern analysis, Metabolic syndrome, Principal component analysis, Reduced rank regression

## Abstract

**Background:**

Dietary patterns were associated with the risk of chronic disease development and outcome-related diseases. In this study, we aimed to compare the correlation between dietary patterns and metabolic syndrome (MetS) using two methods for identifying dietary patterns.

**Methods:**

The participants (*n* = 25,569) aged ≥40 years with impaired kidney function were retrieved from Mei Jau (MJ) Health Screening database from 2008 to 2010. Dietary patterns were identified by principal component analysis (PCA) and reduced rank regression (RRR) from twenty-two food groups using PROC FACTOR and PROC PLS functions.

**Results:**

We identified two similar dietary pattern characteristics (high intakes of deep fried foods, preserved or processed foods, dipping sauce, meat, sugary drinks, organ meats, jam/honey, fried rice/flour products, instant noodles and eggs) derived by PCA and RRR. Logistic regression analysis revealed that RRR-derived dietary pattern scores were positively associated with an odds ratio (OR = 1.70, 95% CI: 1.56, 1.86) of having MetS than PCA-derived dietary pattern scores (OR = 1.38, 95% CI: 1.27, 1.51). The correlations between RRR-derived dietary pattern scores and elevated systolic and diastolic blood pressure (OR = 1.30 for both) or low high density lipoprotein cholesterol in women (OR = 1.32) were statistically significant but not significant in PCA-derived dietary pattern scores.

**Conclusions:**

Our findings suggest that RRR gives better results when studying behavior related dietary patterns in association with MetS. RRR may be more preferable to provide dietary information for developing dietary guidelines among people with MetS. Further studies with prospective measurements are needed to verify whether RRR is a useful analytic tool for the association between dietary patterns and other chronic diseases.

## Background

Chronic disease such as chronic kidney disease and cardiovascular disease has been elevated in the older people, and might be worsened in the presence of metabolic syndrome (MetS) [1]. MetS is defined as a cluster of metabolic disorders characterized by central obesity, dyslipidemia, elevated blood pressure and hyperglycemia [2]. Individuals with MetS were more likely to develop impaired kidney function or the later stage of chronic kidney disease [3]. Previous studies found that the prevalence of metabolic syndrome among dialytic patients in the United States and Finland was 69.3% [4] and 55.7% [5], respectively.

The risk of metabolic syndrome was correlated with dietary intake. Dietary patterns have been used to assess the association between dietary intake and chronic disease [6]. Dietary patterns may provide better information regarding the diet and disease relationship beyond the effects of dietary intake for single nutrient or food [7]. Dietary patterns in relation to MetS were investigated in previous studies. The Western dietary pattern characterized by high intakes of protein, processed foods and refined grains was positively associated with the prevalence of MetS, whereas the healthy dietary pattern with high consumption of vegetables, fruits and dairy products was negatively correlated with MetS [8, 9].

Various methods derived dietary patterns in the epidemiological studies including hypothesis-driven method *(*a priori), data-driven method (a posteriori) or a combination with these two methods [10]. Principal component analysis (PCA), a data-driven method, generates dietary patterns based upon inter correlations between original food intake variables. PCA tends to explain as much variation in dietary intake as possible, and is more likely to represent actual dietary habits in population [11]. However, PCA may have poor correlation with disease risk because behavior-related patterns are not necessarily predictors of the disease of interest [11]. To overcome this issue, a combination method using both a priori and a posteriori approach such as reduced rank regression (RRR) was recently proposed to derive the dietary pattern. This RRR method can explain as much variation in response to disease as possible. Therefore, to compare the dietary patterns derived from PCA with those generated using the RRR method provides more reliable correlation with the disease outcome although the foods in RRR-derived dietary pattern may not be behaviorally associated [12]. A cohort study showed that an increased RRR score was associated with a higher odds ratio of having MetS than an increased PCA pattern in a Northern German population as comparing both PCA and RRR methods [13]. However, the numbers of the participants in the previous study were relatively modest. Therefore, study in the larger population is needed.

Considering that chronic kidney disease has been elevated in the older people, and might be worsened in the presence of MetS, we hypothesized that PCA- and RRR-derived dietary patterns were different in relation to predict the risk of MetS among older people with impaired kidney function. The objective of this study was to investigate and compare the association between dietary patterns and risk of metabolic syndrome among middle-aged and elderly Taiwanese adults with impaired kidney function using both PCA and RRR methods to derive the dietary pattern. In comparison with two different research methodologies, we expected that the dietary pattern derived from RRR method was more strongly associated with MetS among middle-aged and elderly Taiwanese adults with impaired kidney function.

## Methods

### Study participants

The data of the participants with impaired kidney function were retrieved from the database of the Mei Jau (MJ) private health screening centers in Taiwan from 2008 to 2010. The MJ Group has four health screening centers located in Taipei, Taoyuan, Taichung and Kaohsiung, and provides health examination periodically to its members. Participants completed a questionnaire about sociodemographic data, lifestyle and dietary habits prior to anthropometric and biochemical measurements. All participants signed the informed consent authorized by the MJ health screening centers, and the data without personal identification were used for research only. Eligible participants (*n* = 112,140) were aged ≥40 years and had impaired kidney function with estimated glomerular filtration rate (eGFR) < 90 mL/min/1.73 m^2^ and positive urinary protein. We excluded those who had any types of cancer or virus infection (*n* = 48,169), history of any transplantation (*n* = 1765), error values in blood analysis and anthropometric measurements (*n* = 1266), missing data in dietary assessment and other covariates (*n* = 26,605), not complete the questionnaire (*n* = 212) and multiple entries (*n* = 8554). Finally, 25,569 participants were included in the analysis. Taipei Medical University-Joint Institutional Review Board approved this study (TMU-JIRB N201802006).

### Assessment of anthropometric and biochemical variables

Body weight and height were observed by an auto-anthropometer (Nakamura KN-5000A, Tokyo, Japan), and body mass index (BMI) was calculated as the ratio of weight (kg) to the square of height (m^2^). Waist or hip circumference was measured by a flexible tape. Blood pressure was recorded twice at a 10-min interval after resting for 5 min in the sitting position using a standardized sphygmomanometer. Participants were overnight fasting at least for 8 h before a blood test. Uncompensated Jaffe method with alkaline picrate kinetic test was used to measure creatinine levels and eGFR was calculated using the Chronic Kidney Disease Epidemiology Collaboration (CKD-EPI) equation [14]. Meanwhile, urinary protein was measured by Roche Miditron M semi-automated computer-assisted urinalysis system (Combur-10 test M dipstick, Basel, Switzerland). Fasting blood glucose (FBG) and blood lipids such as triglycerides (TG), high density lipoprotein cholesterol (HDL-C), low density lipoprotein cholesterol (LDL-C) and total cholesterol (TC) were analyzed (Toshiba C8000 auto-analyzer, Tokyo, Japan) at the MJ health screening central laboratory. The coefficient of variation for all variables ranged from 1 to 3%. Hypertension and type 2 diabetes were defined as described in the previous study [15]. The definition of hypertension included at least one of the followings: systolic blood pressure ≥ 140 mmHg, diastolic blood pressure ≥ 90 mmHg, use of antihypertensive medication or self-reported hypertension. The definition of diabetes included at least one of the following: (1) FBG ≥ 7.0 mmol/L (≥ 126 mg/dL), (2) use of hypoglycemic medication or (3) self-reported diabetes. The definition of MetS for Asians was to have at least three or more of the followings: (1) waist circumference ≥ 90 cm in men or ≥ 80 cm in women, (2) systolic blood pressure (BP) ≥ 130 mmHg, diastolic BP ≥ 85 mmHg or on anti-hypertensive drug treatment, (3) TG ≥ 1.70 mmol/L (150 mg/dL) or on treatment for lipid abnormality, (4) HDL-C <  1.03 mmol/L (40 mg/dL) in men, < 1.30 mmol/L (50 mg/dL) in women or on treatment for lipid abnormality, (5) FBG ≥ 5.6 mmol/L (100 mg/dL) or on anti-diabetic drug treatment [16].

### Assessment of dietary habits and other covariates

Dietary habits were obtained using standardized and validated self-administered semi quantitative food frequency questionnaire (SQ-FFQ) [17, 18]. Initially, the questionnaire had 85 closed-ended questions on individual food items, twenty-two non-overlapping food groups were classified after standardization and validation as mentioned previously [19]. Participants reported the consumption frequency of each food group on a daily or weekly basis in the past month [19]. The consumption frequency described by the portion size of a bowl, a glass or a serving for one-time intake was categorized into five response options from the lowest to the highest frequency as mentioned previously [19]. The detailed information about the food groups are provided in Additional file 1: Table S1.

Demographic (age, gender, education level, income and marital status) and lifestyle variables (smoking, drinking, sleep quality and physical activity) were recorded using a self-administered questionnaire. Smoking status was classified as ‘yes’ if the participant smoked a cigarette occasionally or daily and as ‘no’ if otherwise. Drinking alcohol was also categorized as ‘no’ (< 1 time/week) and ‘yes’ (≥ 1–2 times/week). Physical activity was assessed by self-reporting intensity (light, moderate and heavy or intense), duration (hours) and frequency (per week) in the last 2 weeks. For sleep quality, participants filled the questions regarding sleep quality and average daily sleep duration in the last month. Sleep quality had five response options (difficulty to fall asleep, difficulty maintaining sleep, feeling of non-restorative sleep, use of sedatives or sleeping pills and no problem to sleep well), and sleep duration had six response options (≤ 4 , 4- < 6, 6- < 7, 7- < 8, 8- < 9 and > 9 h). We defined sleep quality as ‘well’ if the participants had ≥7 h of sleep duration with sleep quality of “no problem to sleep well” and as ‘not well’ if otherwise. For physical activity, the detailed examples of different intensities were described in the self-administered questionnaire. The metabolic equivalent task (MET) for different intensities of physical activity was determined according to previous study [20]. The MET expressed as hours per week was calculated by multiplying the corresponding MET coefficient by duration and frequency of physical activity.

### Statistical analysis

The statistical analysis was performed by SAS 9.4 (SAS Institute Inc., USA) and STATA version 13 (StataCorp LP, College Station, TX, USA). Continuous (non-normal distributed) and categorical variables were presented as median (interquartile range, IQR) and number (percentage), respectively. The characteristics of study subjects with or without MetS were compared using Mann-Whitney or chi-square test for continuous or categorical data, respectively. The multivariable linear regression [β and 95% confidence interval (CI)] and logistic regression (odds ratio (OR) and 95% CI) were used to examine the association of dietary pattern scores with the risk of MetS, components of MetS and their related biomarkers. The *P*-value for trend was analyzed using post-estimation contrast and linear hypothesis test. Moreover, a subgroup analysis based on impaired kidney function categories was used for sensitivity analysis.

### Dietary patterns analysis

**Fig. 1:**
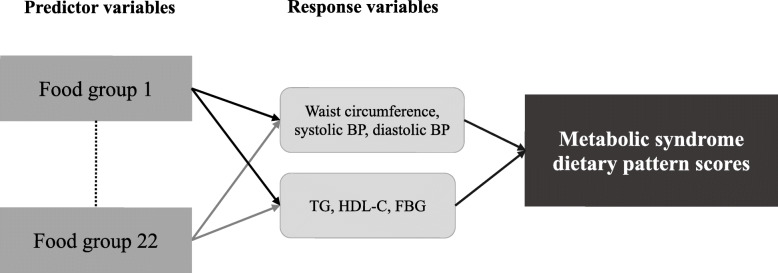
Scheme of metabolic syndrome dietary pattern scores derived by reduced rank regression. BP blood pressure, TG triglycerides, HDL-C high density lipoprotein cholesterol, FBG fasting blood glucose

Dietary patterns were identified by PCA and RRR methods using PROC FACTOR and PROC PLS, respectively. For PCA method, the orthogonal varimax rotation was used and we decided to retain only one from two factors for the comparison. For RRR method, six response variables (waist circumference, TG, HDL-C, systolic BP, diastolic BP and FBG) associated with MetS were used to generate the MetS-specific dietary pattern (Fig. 1). As six response variables were included in the MetS-specific dietary pattern, six factors were generated by RRR method. However, we only retained the first factor that explained the largest percentage (2.4%) of variation in the response variables. The absolute factor loading (Pearson’s correlation coefficient) values ≥0.20 for each food group were the cutoff point to derive the dietary patterns in both PCA and RRR methods. Dietary pattern scores for an individual were calculated by summing intake frequency scores of food groups weighed by their respective factor loading values. However, six food groups had a factor loading ≥0.20 in both PCA-derived dietary patterns. For characterizing the dietary pattern, these food groups could only belong to one factor with a greater factor loading value. Hence, the dietary scores of four food groups (beans/legumes, fried vegetables/salad dressing, rice/flour products and seafood) were neglected in the calculation of the first extracted dietary pattern (Additional file 2: Table S2). For further analysis, dietary pattern scores were divided into quartiles and two adjustment models were performed: model 1 adjusted for age and gender and model 2 adjusted for model 1 variables and education level, income, marital status, smoking, drinking, sleep quality, physical activity and cardiovascular disease status. A *P*-value < 0.05 was considered statistically significant.

## Results

The characteristics of the participants having impaired kidney function with or without MetS are presented in Table 1. The prevalence of MetS was 27.3% (*n* = 6976) and 63.9% (*n* = 4457) of participants with impaired kidney function were male. Participants having impaired kidney function with MetS were likely to be older and have lower kidney function (eGFR 72.0 ± 12.0 vs 75.9 ± 9.6 mL/min/1.73 m^2^, *P* <  0.001, data not shown), less MET, elevated BMI, higher waist-to-hip ratio and worse values for each component of MetS compared with those without MetS. The prevalence of each component of MetS was 24.6% for central obesity, 29.7% for elevated TG, 14.9% for reduced HDL-C, 42.4% for elevated blood pressure and 58.0% for elevated FBG (data not shown). Participants having impaired kidney function with or without MetS statistically differed in the distribution of gender, education level, income, marital status, smoking and drinking. The prevalence of chronic diseases such as type 2 diabetes, hypertension and cardiovascular disease was higher in participants with MetS. Moreover, after adjusting for age, gender, education level, income, marital status, smoking, drinking, sleep quality and physical activity in model 2, participants having impaired kidney function with MetS had an increased risk of having eGFR < 60 mL/min/1.73 m^2^ (OR = 1.64, 95% CI: 1.48, 1.82, *P* <  0.001, data not shown).

**Table 1:** Characteristics of the participants with impaired kidney function by MetS status (*n* = 25,569) ^a^

	All participants (*n* = 25,569)	Participants without MetS (*n* = 18,593)	Participants with MetS (*n* = 6976)	*P* ^b^
Age (years)	51.0 (14.0)	49.0 (13.0)	55.0 (16.0)	< 0.001
Sex, *n* (%)				< 0.001
Male	14,311 (56.0)	9854 (53.0)	4457 (63.9)	
Female	11,258 (44.0)	8739 (47.0)	2519 (36.1)	
Education level, *n* (%)				< 0.001
Below university	16,733 (65.4)	11,748 (63.2)	4985 (71.5)	
University or above	8836 (34.6)	6845 (36.8)	1991 (28.5)	
Income (NTD/year), *n* (%)				< 0.001
< 800,000	11,620 (45.4)	8083 (43.5)	3537 (50.7)	
810,000–1.6 million	9407 (36.8)	7057 (37.9)	2350 (33.7)	
≥ 1.61 million	4542 (17.8)	3453 (18.6)	1089 (15.6)	
Marital status, *n* (%)				< 0.001
No	4050 (15.8)	2822 (15.2)	1228 (17.6)	
Yes	21,519 (84.2)	15,771 (84.8)	5748 (82.4)	
Smoking, *n* (%)				< 0.001
No	21,243 (83.1)	15,660 (84.2)	5583 (80.0)	
Yes	4326 (16.9)	2933 (15.8)	1393 (20.0)	
Drinking alcohol, *n* (%)				< 0.001
No	21,175 (82.8)	15,586 (83.8)	5589 (80.1)	
Yes	4394 (17.2)	3007 (16.2)	1387 (19.9)	
Sleep quality, *n* (%)				0.58
Not well	22,413 (87.7)	16,311 (87.7)	6102 (87.5)	
Well	3156 (12.3)	2282 (12.3)	874 (12.5)	
MET (hours per week)	5.8 (11.7)	5.9 (11.7)	5.8 (11.7)	0.012
BMI (kg/m^2^)	23.8 (4.2)	23.0 (3.6)	26.6 (3.9)	< 0.001
Waist circumference (cm)	80.0 (13.0)	77.0 (12.0)	88.0 (11.0)	< 0.001
Waist-to-hip ratio	0.8 (0.1)	0.8 (0.1)	0.9 (0.1)	< 0.001
Blood pressure (mmHg)				
Systolic	121.0 (24.0)	117.0 (21.0)	134.0 (20.0)	< 0.001
Diastolic	73.0 (16.0)	71.0 (15.0)	80.0 (15.0)	< 0.001
Biomarkers (mmol/L)				
TG	1.2 (0.9)	1.0 (0.7)	1.9 (1.1)	< 0.001
HDL-C	1.4 (0.5)	1.5 (0.5)	1.2 (0.4)	< 0.001
LDL-C	3.1 (1.0)	3.1 (1.0)	3.0 (1.1)	< 0.001
TC	5.2 (1.2)	5.2 (1.1)	5.3 (1.2)	< 0.001
FBG	5.6 (0.7)	5.5 (0.6)	6.0 (0.9)	< 0.001
Creatinine (μmol/L)	91.1 (23.0)	89.3 (23.0)	94.6 (23.9)	< 0.001
eGFR (mL/min/1.73 m^2^)	76.4 (13.5)	77.4 (12.7)	73.4 (15.6)	< 0.001
Urinary protein, *n* (%)				< 0.001
+ 1	24,464 (95.7)	18,133 (97.5)	6331 (90.8)	
+ 2	636 (2.5)	300 (1.6)	336 (4.8)	
≥ + 3	469 (1.8)	160 (0.9)	309 (4.4)	
Prevalence of chronic diseases, *n* (%)				
Type 2 diabetes	2449 (9.6)	832 (4.5)	1617 (23.2)	< 0.001
Hypertension	7450 (29.1)	3220 (17.3)	4230 (60.6)	< 0.001
Cardiovascular disease	1519 (5.9)	749 (4.0)	770 (11.0)	< 0.001

*MetS* metabolic syndrome, *NTD* New Taiwan dollar, *MET* metabolic equivalent task, *BMI* body mass index, *TG* triglycerides, *HDL-C* high density lipoprotein cholesterol, *LDL-C* low density lipoprotein cholesterol, *TC* total cholesterol, *FBG* fasting blood glucose, *eGFR* estimated glomerular filtration rate

^a^Continuous variables are presented as median (interquartile range). Categorical variables are presented as absolute frequency (percentage)

^b^The *P*-values were tested using Mann-Whitney test for continuous variables and chi-square test for categorical variables

### Dietary pattern analysis

**Fig. 2:**
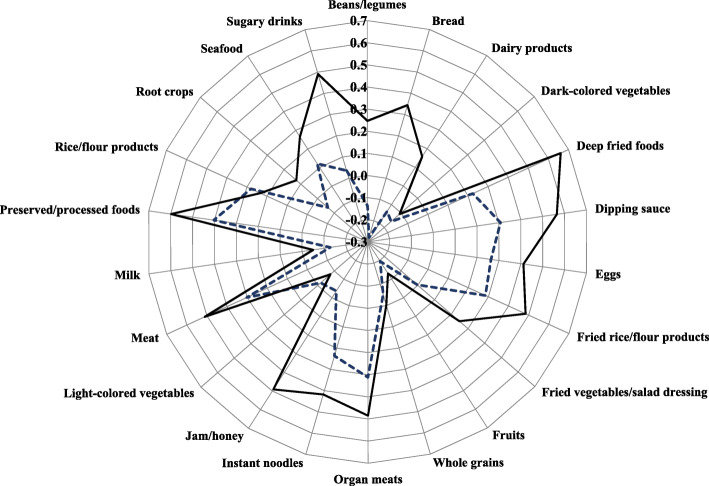
Radar chart of Pearson’s correlation coefficients between food groups and two dietary patterns. The dietary patterns were derived by principal component analysis (—) and reduced rank regression (−--)

Pearson’s correlation coefficients between food groups and both PCA- and RRR-derived dietary patterns are shown in Fig. 2. For the purpose of comparison, we only considered the first extraction pattern in both PCA and RRR methods because the first extraction pattern explained the most variations in predictor variables (food groups) in PCA-derived dietary pattern or response variables in RRR-derived dietary pattern. Moreover, the first pattern from both methods had relatively same characteristics, which generates easier interpretation. The first dietary pattern (fried-processed dietary pattern) derived by PCA method was characterized by frequent intake of deep fried foods, preserved or processed foods, dipping sauce, meat, sugary drinks, organ meats, jam or honey, fried rice or flour products, instant noodles and eggs. The pattern derived by RRR method seemed to have similar characteristics (high intakes in processed foods, organ meats, dipping sauce, meat, fried rice or flour products, rice or flour products, eggs, instant noodles and deep fried foods, but low intakes in fruits and bread) with PCA-derived dietary pattern. As expected, the percentage of variation explained by food groups or predictors was higher in PCA-derived dietary pattern compared with that in RRR-derived dietary pattern (15.8% vs 6.9% respectively). The RRR-derived dietary pattern explained 2.4% of the cumulative variation in six response variables and mainly driven by the explained variation in waist circumference (5.8%) and TG (2.4%). The detailed information of correlation coefficient and variation explained in both methods are provided in Additional file 2: Table S2.

### Association between dietary patterns and metabolic syndrome

The association between PCA- or RRR-derived dietary pattern scores and MetS are presented in Table 2. The number of the participants having impaired kidney function with MetS increased across the increasing quartiles of RRR-derived dietary pattern scores. However, the number of the participants having impaired kidney function with MetS was greater in quartile 1 (Q1) and quartile 4 (Q4) of PCA-derived dietary pattern scores. Compared with the participants with impaired kidney function in the reference group (Q1), those in higher quartiles (Q3 and Q4) of PCA- or RRR-derived dietary pattern scores had 1.11–1.37 or 1.30–1.70 times higher odds ratios of having MetS, respectively, after adjustment for potential confounders in model 2. The association between PCA- or RRR-derived dietary pattern scores and components of MetS is illustrated in Tables 3 and 4. Participants with impaired kidney function in Q4 of PCA-derived dietary pattern scores were significantly associated with 1.64 (95% CI: 1.46, 1.84), 1.79 (95% CI: 1.56, 2.05), 1.25 (95% CI: 1.15, 1.35) and 1.21 (95% CI: 1.12, 1.31) times increased risk of having high waist circumference in men and women, elevated TG and elevated FBG, respectively, compared with those in Q1 (Table 3). Compared with the participants with impaired kidney function in Q1, those in Q4 of RRR-derived dietary pattern scores had 1.88 (95% CI: 1.67, 2.11), 2.14 (95% CI: 1.87, 2.46), 1.46 (95% CI: 1.35, 1.59) and 1.37 (95% CI: 1.27, 1.48) times higher odds ratios of having high waist circumference in men and women, elevated TG and elevated FBG, respectively (Table 4). Moreover, the association of RRR-derived dietary pattern scores with elevated systolic or diastolic BP (OR = 1.30 for both) and lower HDL-C in women (OR = 1.31; 95% CI: 1.13, 1.52) was statistically significant. However, the correlations of PCA-derived dietary pattern scores with elevated BP and lower HDL-C in women were not significant. After adjustment for potential confounders, PCA-derived dietary pattern scores had a linear association with all anthropometric data, biochemical parameters and blood pressure except diastolic BP (Additional file 3: Table S3). As expected, RRR-derived dietary pattern scores had stronger linear association with all anthropometric data, biochemical parameters and blood pressure compared with PCA-derived dietary pattern scores. Moreover, a subgroup analysis shown a consistent results that RRR-derived dietary pattern had stronger association with risk of MetS compared with PCA-derived dietary pattern regardless of impaired kidney function categories (Additional file 4: Table S4).

**Table 2:** Association of PCA- or RRR-derived dietary pattern with MetS across quartiles of dietary pattern scores ^a^

	Quartiles (Q) of dietary pattern scores (*n* = 25,569)				*P*-trend
	Q1	Q2	Q3	Q4	
	OR	OR (95% CI)	OR (95% CI)	OR (95% CI)	
PCA-derived dietary pattern					
Total (*n*)	6418	6365	6392	6394	
MetS (*n*)	1762	1652	1665	1897	
Model 1	1	1.02 (0.94, 1.11)	1.11 (1.02, 1.20)	1.40 (1.29, 1.53)	< 0.001
Model 2	1	1.03 (0.95, 1.12)	1.11 (1.02, 1.21)	1.37 (1.26, 1.49)	< 0.001
RRR-derived dietary pattern					
Total (*n*)	6403	6332	6468	6366	
MetS (*n*)	1495	1582	1793	2106	
Model 1	1	1.11 (1.02, 1.21)	1.33 (1.22, 1.44)	1.78 (1.64, 1.93)	< 0.001
Model 2	1	1.11 (1.02, 1.20)	1.30 (1.20, 1.41)	1.70 (1.56, 1.85)	< 0.001

*PCA* principal component analysis, *RRR* reduced rank regression, *MetS* metabolic syndrome

^a^Data are presented as odds ratios (ORs) and 95% confidence intervals (95% CIs). Model 1: adjusted for age and gender. Model 2: adjusted for age, gender, education level, income, marital status, smoking, drinking, sleep quality, physical activity and cardiovascular disease status

**Table 3:** Association of PCA-derived dietary pattern with components of MetS across quartiles of dietary pattern scores ^a^

	Quartiles (Q) of PCA-derived dietary pattern scores (*n* = 25,569)				*P*-trend
	Q1 (*n* = 6418)	Q2 (*n* = 6365)	Q3 (*n* = 6392)	Q4 (*n* = 6394)	
	OR	OR (95% CI)	OR (95% CI)	OR (95% CI)	
High waist circumference in men					
Model 1	1	1.07 (0.95, 1.20)	1.29 (1.15, 1.45)	1.72 (1.54, 1.93)	< 0.001
Model 2	1	1.05 (0.93, 1.19)	1.27 (1.12, 1.42)	1.64 (1.46, 1.84)	< 0.001
High waist circumference in women					
Model 1	1	1.07 (0.95, 1.22)	1.26 (1.11, 1.44)	1.78 (1.55, 2.04)	< 0.001
Model 2	1	1.08 (0.95, 1.22)	1.28 (1.12, 1.45)	1.79 (1.56, 2.05)	< 0.001
Elevated systolic BP					
Model 1	1	0.97 (0.90, 1.05)	0.99 (0.91, 1.07)	1.03 (0.95, 1.12)	0.383
Model 2	1	0.98 (0.91, 1.06)	1.00 (0.92, 1.08)	1.04 (0.96, 1.13)	0.261
Elevated diastolic BP					
Model 1	1	0.92 (0.84, 1.01)	0.95 (0.86, 1.04)	1.01 (0.92, 1.11)	0.682
Model 2	1	0.93 (0.85, 1.02)	0.95 (0.87, 1.04)	1.02 (0.93, 1.12)	0.579
Elevated TG					
Model 1	1	1.02 (0.94, 1.11)	1.12 (1.03, 1.21)	1.30 (1.20, 1.41)	< 0.001
Model 2	1	1.02 (0.94, 1.10)	1.10 (1.02, 1.20)	1.25 (1.15, 1.35)	< 0.001
Low HDL-C in men					
Model 1	1	0.99 (0.86, 1.15)	1.03 (0.89, 1.19)	1.06 (0.92, 1.22)	0.346
Model 2	1	0.98 (0.84, 1.14)	1.03 (0.89, 1.19)	1.03 (0.90, 1.20)	0.506
Low HDL-C in women					
Model 1	1	1.02 (0.90, 1.16)	0.91 (0.79, 1.05)	1.11 (0.96, 1.29)	0.394
Model 2	1	1.02 (0.90, 1.17)	0.92 (0.80, 1.06)	1.13 (0.97, 1.31)	0.302
Elevated FBG					
Model 1	1	1.06 (0.99, 1.14)	1.02 (0.95, 1.10)	1.21 (1.12, 1.30)	< 0.001
Model 2	1	1.07 (0.99, 1.15)	1.03 (0.95, 1.11)	1.21 (1.12, 1.31)	< 0.001

*PCA* principal component analysis, *MetS* metabolic syndrome, *BP* blood pressure, *TG* triglycerides, *HDL-C* high density lipoprotein cholesterol, *FBG* fasting blood glucose

^a^The odds ratios across quartiles of dietary pattern scores were compared with the reference group (Q1). Components of metabolic syndrome were defined as follows: high waist circumference (≥ 90 cm in men or ≥ 80 cm in women), elevated systolic BP (≥ 130 mmHg), elevated diastolic BP (≥ 85 mmHg), elevated TG (≥ 1.70 mmol/L), low HDL-C (< 1.03 mmol/L in men or <  1.30 mmol/L in women) and elevated FBG (≥ 5.60 mmol/L). Model 1: adjusted for age and gender (except waist circumference and HDL-C). Model 2: adjusted for age, gender (except waist circumference and HDL-C), education level, income, marital status, smoking, drinking, sleep quality, physical activity and cardiovascular disease status

**Table 4:** Association of RRR-derived dietary pattern with components of MetS across quartiles of dietary pattern scores ^a^

	Quartiles (Q) of RRR-derived dietary pattern scores (*n* = 25,569)				*P*-trend
	Q1 (*n* = 6403)	Q2 (*n* = 6332)	Q3 (*n* = 6468)	Q4 (*n* = 6366)	
	OR	OR (95% CI)	OR (95% CI)	OR (95% CI)	
High waist circumference in men					
Model 1	1	1.17 (1.03, 1.32)	1.27 (1.13, 1.43)	2.00 (1.78, 2.24)	< 0.001
Model 2	1	1.16 (1.02, 1.31)	1.23 (1.09, 1.39)	1.88 (1.67, 2.11)	< 0.001
High waist circumference in women					
Model 1	1	1.09 (0.96, 1.24)	1.68 (1.48, 1.90)	2.20 (1.92, 2.53)	< 0.001
Model 2	1	1.08 (0.95, 1.22)	1.65 (1.45, 1.87)	2.14 (1.87, 2.46)	< 0.001
Elevated systolic BP					
Model 1	1	1.03 (0.96, 1.12)	1.14 (1.05, 1.23)	1.32 (1.22, 1.43)	< 0.001
Model 2	1	1.03 (0.95, 1.11)	1.13 (1.04, 1.22)	1.30 (1.20, 1.41)	< 0.001
Elevated diastolic BP					
Model 1	1	1.04 (0.95, 1.14)	1.10 (1.01, 1.21)	1.32 (1.21, 1.45)	< 0.001
Model 2	1	1.04 (0.94, 1.14)	1.10 (1.00, 1.20)	1.30 (1.19, 1.43)	< 0.001
Elevated TG					
Model 1	1	1.09 (1.01, 1.18)	1.28 (1.18, 1.38)	1.56 (1.44, 1.69)	< 0.001
Model 2	1	1.08 (0.99, 1.17)	1.24 (1.14, 1.34)	1.46 (1.35, 1.59)	< 0.001
Low HDL-C in men					
Model 1	1	0.96 (0.83, 1.11)	1.00 (0.87, 1.16)	1.06 (0.92, 1.21)	0.364
Model 2	1	0.96 (0.83, 1.12)	0.99 (0.86, 1.15)	1.04 (0.90, 1.21)	0.482
Low HDL-C in women					
Model 1	1	1.13 (0.99, 1.29)	1.14 (0.99, 1.30)	1.31 (1.13, 1.52)	< 0.001
Model 2	1	1.13 (0.99, 1.28)	1.13 (0.98, 1.29)	1.31 (1.13, 1.52)	< 0.001
Elevated FBG					
Model 1	1	1.11 (1.04, 1.20)	1.19 (1.10, 1.28)	1.37 (1.27, 1.48)	< 0.001
Model 2	1	1.12 (1.04, 1.20)	1.18 (1.10, 1.28)	1.37 (1.27, 1.48)	< 0.001

*RRR* reduced rank regression, *MetS* metabolic syndrome, *BP* blood pressure, *TG* triglycerides, *HDL-C* high density lipoprotein cholesterol, *FBG* fasting blood glucose

^a^The odds ratios across quartiles of dietary pattern scores were compared with the reference group (Q1). Components of metabolic syndrome were defined as follows: high waist circumference (≥ 90 cm in men or ≥ 80 cm in women), elevated systolic BP (≥ 130 mmHg), elevated diastolic BP (≥ 85 mmHg), elevated TG (≥ 1.70 mmol/L), low HDL-C (< 1.03 mmol/L in men or < 1.30 mmol/L in women) and elevated FBG (≥ 5.60 mmol/L). Model 1: adjusted for age and gender (except waist circumference and HDL-C). Model 2: adjusted for age, gender (except waist circumference and HDL-C), education level, income, marital status, smoking, drinking, sleep quality, physical activity and cardiovascular disease status

## Discussion

Our data supported a potential association between dietary patterns and the prevalence of MetS among middle-aged and elderly adults with impaired kidney function using two different methods to derive dietary patterns. Both PCA and RRR methods produced similar dietary patterns. The dietary pattern derived by PCA method reflected dietary behavior, and the dietary pattern identified by RRR method is more likely to have a diet-disease association. The similar dietary pattern was obtained from these two methods indicating RRR-derived dietary pattern also reflects the eating behavior of the population [13]. In addition, the RRR-derived dietary pattern was more strongly associated with response variables than the PCA-derived dietary pattern. Consistent with our findings, previous studies also observed the same results in the association between dietary patterns and cardiovascular risk factors [21, 22] or all-cause mortality [23] among middle-aged and/or elderly adults. In addition, the RRR-derived dietary pattern had a stronger correlation with markers of subclinical atherosclerosis compared with the PCA-derived dietary pattern among multi-ethnic middle-aged and elderly adults in the United States [24]. The Growth, Exercise and Nutrition Epidemiological Study in preSchoolers (GENESIS) study also revealed that the RRR-derived dietary pattern showed a significant association with childhood obesity among Greek preschool children, but the PCA-derived dietary pattern did not have any correlation [25]. The previous studies within the framework of the Study on the Epidemiology of Psychological, Alimentary Health and Nutrition (SEPAHAN) explored the association between dietary patterns derived by PCA [26] or RRR method [27] and psychological disorders among Iranian adults. The RRR-derived healthy dietary pattern with high intake of whole grains, low-fat dairy products, vegetables, fruits and nuts had better association with a lower risk of psychological disorders compared with the PCA-derived lacto-vegetarian dietary pattern [26, 27].

The main advantage of using RRR method to establish the dietary pattern is to incorporate the prior knowledge with better explanation of response variables rather than only revealing the general eating pattern in population [22]. Therefore, in most studies, RRR-derived dietary patterns were associated with disease of interest, but not necessarily reflected real-world dietary pattern [13]. In the present study, participants with high adherence to a RRR-derived dietary pattern had higher OR of having MetS compared with those with high adherence to a PCA-derived dietary pattern. Moreover, the linear regression analysis showed β coefficients corresponding to RRR method were stronger than corresponding to PCA method. Since RRR method explicitly derives the predictors which explain the maximum of response variables, the dietary pattern identified by RRR is more likely to be closely associated with health outcomes compared with that derived by PCA [23, 28]. This argument was supported by the finding of Naja et al. [29], and the data showed that the dietary pattern derived by RRR was correlated with a higher OR of elevated BP than that derived by PCA among Lebanese adult men. This finding could be attributed to the fact that the RRR-derived dietary pattern explained more variation in response variables than the PCA-derived dietary pattern [29].

Consistent with previous studies [13, 24, 30], we used components of MetS as response variables in RRR method. In our study, RRR-derived dietary pattern was characterized by high intakes of preserved or processed foods, deep fried foods, meat and sugary drinks but low intakes of fruits and bread, which was similar to Western or unhealthy dietary pattern found in previous studies [7, 28, 31–33]. Indeed, participants with high adherence to this dietary pattern had a positive association with the prevalence of MetS. Although the lower but significant correlations of PCA-derived dietary pattern with components of MetS compared with that of RRR-derived dietary pattern, the similar results were found regarding the association between PCA- or RRR-derived dietary pattern and components of MetS. In consistent with the previous findings using PCA method to derive the dietary pattern, a Western-type dietary pattern with high intake of white bread, processed meat, fries, hamburger, hot dog and salty snacks was associated with a higher risk of developing MetS [34]. In the present study, both PCA- and RRR-derived dietary patterns showed significant correlations with all metabolic components, but PCA-derived dietary pattern was not associated with diastolic BP.

Several possible mechanisms may explain the linear effect of unhealthy dietary pattern and the components of MetS. The food components of this dietary pattern such as preserved or processed foods, deep fried foods, meat and sugary drinks plausibly contribute to an increased risk of MetS. This dietary pattern was often accompanied by high intakes of total fat, saturated fat and simple sugar which may stimulate the production and secretion of certain pro-inflammatory cytokines including C-reactive protein (CRP) and further increase systemic inflammation [24, 30, 35]. The pro-inflammatory cytokine CRP has been known to be closely related to inflammation and MetS [36]. Our previous study demonstrated that participants who consumed high intake of a Western-type dietary pattern had increased odds of components of MetS and CRP [37]. The plausible mechanisms for the effect of CRP on the increased risk of MetS included the impairment of insulin signaling pathway and pro-atherogenic effects on vascular cells [38]. Chronic inflammation was associated with insulin resistance, dyslipidemia and elevated BP [29, 35]. Participants with higher adherence to a Western diet tended to have higher prevalence of hypertension, which may be partially correlated with high intakes of fat and protein from animal food sources in this particular dietary pattern [39, 40]. The International Study on Macro/Micronutrients and Blood Pressure (INTERMAP) study also reported a significant linear association between total protein intake and blood pressure [41]. Additionally, high consumption of red meat dietary pattern could be correlated with deposition of iron, particularly heme-iron. Subjects in the MetS group had an elevated iron overload than those in the age-matched control group [42]. Therefore, high iron contents in red meat might be related to an increased prevalence of MetS [42–45]. A recent meta-analysis study indicated that adherence to a posteriori meat/Western dietary pattern characterized by high intakes of meat, processed foods and fast foods significantly increased risk of MetS by 19% [33]. Similarly, other studies found that meat/Western dietary pattern was associated with an increased risk of MetS by 16% [31] to 28% [32].

The major strength of our study was the use of different approaches to derive dietary patterns and comparison of these two results. The similar dietary patterns obtained from PCA and RRR methods might indicate dietary behavior, which was assumed to be on the causal pathway from dietary pattern to the disease of interest. Furthermore, we had a large study population that could describe the dietary pattern in a greater scale. However, several limitations in the present study should be considered. First, the limitation of the study was the cross-sectional design, which made it difficult to have causal inference. Secondly, in corresponding with a previous study [32], many possible confounding factors (demographic and lifestyle factors) had been controlled in the present study, yet we were not able to control other factors such as total energy and protein intake, family history of diabetes, hypertension or cardiovascular disease and intake of other drugs. These unmeasured factors may have introduced residual confounding. Lastly, although the questionnaire in this study had been validated for Taiwanese population, dietary intake was assessed using self-administered SQ-FFQ and under-reporting may occur.

## Conclusion

In summary, both PCA and RRR methods obtain a similar dietary pattern which is associated with components of MetS among middle-aged and elderly adults with impaired kidney function. This similarity allows to assess the likeness between real eating behavior and MetS-related dietary patterns. Even though both dietary patterns have a linear association with components of MetS, RRR method shows stronger statistical correlations. Therefore, RRR method may be more suitable to evaluate dietary information for designing and realizing dietary guidelines. Moreover, adequate dietary intake in people with impaired kidney function is important to manage kidney disease, and designing dietary guidelines based on dietary pattern analysis by incorporating kidney function biomarkers as the response variables is necessary to prevent the severity of kidney disease. However, further research is needed to confirm the association between RRR-derived dietary pattern and other disease outcomes in combination with prospective measurements.

## Supplementary information

**Additional file 1: Table S1.** Food groups used in the dietary pattern analysis.

**Additional file 2: Table S2–1.** Factor loadings **(**Pearson’s correlation coefficients) of food groups in PCA-derived dietary patterns ^a^. **Table S2–2.** Percentage of variation and Pearson’s correlation coefficients among food groups and response variables in RRR-derived dietary pattern.

**Additional file 3: Table S3.** Linear associations of PCA- or RRR-derived dietary pattern scores with components of MetS.

**Additional file 4: Table S4.** Association of PCA- or RRR-derived dietary pattern with MetS in subgroups by impaired kidney function ^a^

## Data Availability

The data that support the findings of this study are available from Mei Jau (MJ) Health Institute, but restricted for research use only. The data are not publicly available. Data are available from the authors upon reasonable request and with permission of MJ Health Institute.

## Abbreviations

BMI: Body mass index
BP: Blood pressure
eGFR: Estimated glomerular filtration rate
FBG: Fasting blood glucose
HDL-C: High density lipoprotein cholesterol
LDL-C: Low density lipoprotein cholesterol
MET: Metabolic equivalent task
MetS: Metabolic syndrome
MJ: Mei Jau
PCA: Principal component analysis
RRR: Reduced rank regression
SQ-FFQ: Food frequency questionnaire
TC: Total cholesterol
TG: Triglycerides
