# Supplementary material for: Comparing two methods for deriving dietary patterns associated with risk of metabolic syndrome among middle-aged and elderly Taiwanese adults with impaired kidney function
Source: BMC Med Res Methodol. 2020 Oct 14;20:255. doi: 10.1186/s12874-020-01142-4 (PMC7559471; doi:10.1186/s12874-020-01142-4)
Supplement: Supplementary file 1 — Additional file 1: Table S1. Food groups used in the dietary pattern analysis. [file 12874_2020_1142_MOESM1_ESM.docx]

**Comparing two methods for deriving dietary patterns associated with risk of metabolic syndrome among middle-aged and elderly Taiwanese adults with impaired kidney function**

Adi Lukas Kurniawan^1^, Chien-Yeh Hsu^2,3^, Hsiu-An Lee^4^, Hsiao-Hsien Rau^5^, Rathi Paramastri^1^, Ahmad Syauqy^1,6^ and Jane C.-J. Chao^1,3,7*^

**Table S1** Food groups used in the dietary pattern analysis

| Food group | Food item examples |
| --- | --- |
| Rice/flour products | Rice, flour, noodles, bread, baked wheat bread or twisted cruller |
| Whole grains | Whole wheat bread, brown rice, mixed grains or oatmeal |
| Fried rice/flour products | Fried rice or fried noodles |
| Root crops | Sweet potato, potato, taro or corn |
| Bread | Red bean bread, butter bread, cake or cookies |
| Beans/legumes | Packaged tofu, soybean milk or dried bean curd |
| Seafood | Fish, row fish, shrimps or oysters |
| Eggs | Chicken egg, duck egg or quail egg |
| Meat | Pork, beef, lamb, veal, chicken, duck or goose |
| Organ meats | Liver, heart, kidneys or intestines |
| Milk | Fresh milk, drying yogurt or powdered milk |
| Dairy products | Yogurt or cheese |
| Light-colored vegetables | Cabbage, pechay, cucumber, squash or radish |
| Dark-colored vegetables | Carrot, green leafy vegetables or tomato |
| Fried vegetables/salad dressing | Cooking in oil or salad dressing |
| Fruits | Apple, dragon fruit, grapes, grapefruit, guava, kiwi, lychees, orange, papaya or watermelon |
| Deep fried foods | All foods that are deep fried in oil |
| Jam/honey | Jam or honey |
| Sugary drinks | Soft drinks, coffee/tea with sugar, mung bean paste or fruit juice |
| Preserved/processed foods | Ham, sausage, canned foods, preserved vegetables or preserved fish |
| Instant noodles | Instant noodles |
| Dipping sauce | Soy sauce, ketchup, hot sauce, vinegar or pepper salt |
