# Supplementary material for: Comparing two methods for deriving dietary patterns associated with risk of metabolic syndrome among middle-aged and elderly Taiwanese adults with impaired kidney function
Source: BMC Med Res Methodol. 2020 Oct 14;20:255. doi: 10.1186/s12874-020-01142-4 (PMC7559471; doi:10.1186/s12874-020-01142-4)
Supplement: Supplementary file 2 — Additional file 2: Table S2–1. Factor loadings (Pearson’s correlation coefficients) of food groups in PCA-derived dietary patterns a. Table S2–2. Percentage of variation and Pearson’s correlation coefficients among food groups and response variables in RRR-derived dietary pattern. [file 12874_2020_1142_MOESM2_ESM.docx]

**Comparing two methods for deriving dietary patterns associated with risk of metabolic syndrome among middle-aged and elderly Taiwanese adults with impaired kidney function**

Adi Lukas Kurniawan^1^, Chien-Yeh Hsu^2,3^, Hsiu-An Lee^4^, Hsiao-Hsien Rau^5^, Rathi Paramastri^1^, Ahmad Syauqy^1,6^ and Jane C.-J. Chao^1,3,7*^

**Table S2-1** Factor loadings **(**Pearson’s correlation coefficients) of food groups in PCA-derived dietary patterns ^a^

| Food group | Fried-processed dietary pattern  (Pattern 1) | Vege-fruits dietary pattern  (Pattern 2) |
| --- | --- | --- |
| Beans/legumes ^b^ | 0.247 | **0.456** |
| Bread | **0.344** | 0.176 |
| Dairy products | 0.158 | **0.296** |
| Dark-colored vegetables | -0.108 | **0.771** |
| Deep fried foods | **0.661** | 0.029 |
| Dipping sauce | **0.565** | -0.015 |
| Eggs ^b^ | **0.412** | 0.213 |
| Fried rice/flour products | **0.485** | 0.103 |
| Fried vegetables/salad dressing ^b^ | 0.248 | **0.38** |
| Fruits | -0.130 | **0.553** |
| Whole grains | -0.002 | **0.371** |
| Organ meats | **0.485** | 0.065 |
| Instant noodles | **0.418** | -0.1 |
| Jam/honey | **0.491** | -0.001 |
| Light-colored vegetables | -0.077 | **0.752** |
| Meat | **0.511** | 0.209 |
| Milk | -0.051 | **0.259** |
| Preserved/processed foods | **0.598** | 0.03 |
| Rice/flour products ^b^ | 0.233 | **0.236** |
| Root crops | 0.126 | **0.521** |
| Seafood ^b^ | 0.265 | **0.398** |
| Sugary drinks | **0.491** | -0.133 |
| **Explained variance extracted from pattern (%)** | **15.8** | **10.9** |

^a^ Beans/legumes, eggs, fried vegetables/salad dressing, meat, rice/flour products and seafood had a factor loading ≥ 0.20 in both PCA-derived dietary patterns. However, for the characteristics of the dietary pattern, the food group could only belong to one factor with a greater factor loading value.

^b^ The dietary scores of beans/legumes, fried vegetables/salad dressing, rice/flour products and seafood were neglected in the calculation of the first extracted dietary pattern.

**Table S2-2** Percentage of variation and Pearson’s correlation coefficients among food groups and response variables in RRR-derived dietary pattern

|  | Percentage of variation (%) | Pearson’s correlation coefficient |
| --- | --- | --- |
| **Food group** |  |  |
| Beans/legumes | 2.86 | -0.14 |
| Bread | 11.87 | -0.28 |
| Dairy products | 2.84 | -0.14 |
| Dark-colored vegetables | 3.87 | -0.16 |
| Deep fried foods | 7.61 | 0.22 |
| Dipping sauce | 14.38 | 0.31 |
| Eggs | 10.58 | 0.26 |
| Fried rice/flour products | 12.37 | 0.29 |
| Fried vegetables/salad dressing | 0.00 | 0.00 |
| Fruits | 5.84 | -0.20 |
| Whole grains | 0.42 | -0.05 |
| Organ meats | 14.77 | 0.31 |
| Instant noodles | 8.51 | 0.24 |
| Jam/honey | 0.21 | -0.04 |
| Light-colored vegetables | 0.05 | -0.02 |
| Meat | 13.85 | 0.30 |
| Milk | 2.53 | -0.13 |
| Preserved/processed foods | 24.68 | 0.40 |
| Rice/flour products | 11.92 | 0.28 |
| Root crops | 0.59 | -0.06 |
| Seafood | 2.23 | 0.12 |
| Sugary drinks | 0.20 | 0.04 |
| **Explained variance extracted from pattern** | **6.92** |  |
| **Explained variance in response variables** | **2.35** |  |
| Waist circumference | 5.84 | 0.64 |
| Systolic BP | 1.00 | 0.27 |
| Diastolic BP | 1.73 | 0.35 |
| TG | 2.36 | 0.41 |
| HDL-C | 2.19 | -0.39 |
| FBG | 1.00 | 0.27 |

BP blood pressure, TG triglycerides, HDL-C high density lipoprotein cholesterol, FBG fasting blood glucose.
