# Supplementary material for: Comparing two methods for deriving dietary patterns associated with risk of metabolic syndrome among middle-aged and elderly Taiwanese adults with impaired kidney function
Source: BMC Med Res Methodol. 2020 Oct 14;20:255. doi: 10.1186/s12874-020-01142-4 (PMC7559471; doi:10.1186/s12874-020-01142-4)
Supplement: Supplementary file 3 — Additional file 3: Table S3. Linear associations of PCA- or RRR-derived dietary pattern scores with components of MetS. [file 12874_2020_1142_MOESM3_ESM.docx]

**Comparing two methods for deriving dietary patterns associated with risk of metabolic syndrome among middle-aged and elderly Taiwanese adults with impaired kidney function**

Adi Lukas Kurniawan^1^, Chien-Yeh Hsu^2,3^, Hsiu-An Lee^4^, Hsiao-Hsien Rau^5^, Rathi Paramastri^1^, Ahmad Syauqy^1,6^ and Jane C.-J. Chao^1,3,7*^

**Table S3** Linear associations of PCA- or RRR-derived dietary pattern scores with components of MetS

| Variables | PCA-derived dietary pattern ^a^ | | | | RRR-derived dietary pattern ^b^ | | | |
| --- | --- | --- | --- | --- | --- | --- | --- | --- |
|  | Model 1 ^c^ | | Model 2 ^d^ | | Model 1 ^c^ | | Model 2 ^d^ | |
|  | β (95% CI) | | β (95% CI) | | β (95% CI) | | β (95% CI) | |
| BMI (kg/m^2^) | 0.17 (0.15, 0.19) | | 0.17 (0.15, 0.19) | | 0.52 (0.48, 0.56) | | 0.50 (0.46, 0.55) | |
| Waist circumference (cm) |  |  |  |  |  |  |  |  |
| Men | 0.48 (0.41, 0.54) | | 0.45 (0.38, 0.51) | | 1.23 (1.09, 1.37) | | 1.17 (1.02, 1.31) | |
| Women | 0.50 (0.43, 0.57) | | 0.50 (0.42, 0.57) | | 1.55 (1.39, 1.71) | | 1.50 (1.33, 1.67) | |
| Systolic BP (mmHg) | 0.14 (0.03, 0.24) | | 0.16 (0.06, 0.26) | | 1.21 (0.98, 1.43) | | 1.16 (0.93, 1.39) | |
| Diastolic BP (mmHg) | 0.04 (-0.03, 0.11) | | 0.05 (-0.01, 0.12) | | 0.73 (0.58, 0.88) | | 0.70 (0.55, 0.85) | |
| TG (mmol/L) | 0.03 (0.02, 0.03) | | 0.02 (0.01, 0.03) | | 0.10 (0.09, 0.11) | | 0.08 (0.07, 0.09) | |
| HDL-C (mmol/L) |  |  |  |  |  |  |  |  |
| Men | -0.01 (-0.01, -0.00) | | -0.00 (-0.01, -0.00) | | -0.01 (-0.02, -0.00) | | -0.01 (-0.02, -0.00) | |
| Women | -0.01 (-0.01, -0.00) | | -0.01 (-0.01, -0.00) | | -0.03 (-0.04, -0.02) | | -0.03 (-0.04, -0.02) | |
| LDL-C (mmol/L) | 0.02 (0.01, 0.03) | | 0.02 (0.01, 0.02) | | 0.03 (0.02, 0.04) | | 0.03 (0.02, 0.04) | |
| TC (mmol/L) | 0.02 (0.02, 0.03) | | 0.02 (0.02, 0.03) | | 0.05 (0.03, 0.06) | | 0.04 (0.03, 0.05) | |
| FBG (mmol/L) | 0.01 (0.01, 0.02) | | 0.01 (0.01, 0.02) | | 0.09 (0.07, 0.11) | | 0.08 (0.07, 0.10) | |

PCA principal component analysis, RRR reduced rank regression, MetS metabolic syndrome, BMI body mass index, BP blood pressure, TG triglycerides, HDL-C high density lipoprotein cholesterol, LDL-C low density lipoprotein cholesterol, TC total cholesterol, FBG fasting blood glucose.

^a^ The *P*-value for all < 0.01 except diastolic BP (*P* = 0.1).

^b^ The *P*-value for all < 0.001.

^c^ Adjusted for age and gender (except waist circumference and HDL-C).

^d^ Adjusted for age, gender (except waist circumference and HDL-C), education level, income, marital status, smoking, drinking, sleep quality, physical activity and cardiovascular disease status.
