# Supplementary material for: Comparing two methods for deriving dietary patterns associated with risk of metabolic syndrome among middle-aged and elderly Taiwanese adults with impaired kidney function
Source: BMC Med Res Methodol. 2020 Oct 14;20:255. doi: 10.1186/s12874-020-01142-4 (PMC7559471; doi:10.1186/s12874-020-01142-4)
Supplement: Supplementary file 4 — Additional file 4: Table S4. Association of PCA- or RRR-derived dietary pattern with MetS in subgroups by impaired kidney function a [file 12874_2020_1142_MOESM4_ESM.docx]

**Comparing two methods for deriving dietary patterns associated with risk of metabolic syndrome among middle-aged and elderly Taiwanese adults with impaired kidney function**

Adi Lukas Kurniawan^1^, Chien-Yeh Hsu^2,3^, Hsiu-An Lee^4^, Hsiao-Hsien Rau^5^, Rathi Paramastri^1^, Ahmad Syauqy^1,6^ and Jane C.-J. Chao^1,3,7*^

**Table S4** Association of PCA- or RRR-derived dietary pattern with MetS in subgroups by impaired kidney function ^a^

|  | Quartiles (Q) of dietary pattern scores (*n* = 25,569) | | | | | | | *P*-trend |
| --- | --- | --- | --- | --- | --- | --- | --- | --- |
|  | Q1 | Q2 | | Q3 | | Q4 | |  |
|  | OR | OR (95% CI) | | OR (95% CI) | | OR (95% CI) | |  |
| Participants with mild impaired kidney function (eGFR 60-90 mL/min/1.73 m^2^) | | | | | | | |  |
| PCA-derived dietary pattern |  |  |  |  |  |  |  |  |
| Total (*n*) | 5739 | 5790 | | 5921 | | 6009 | |  |
| MetS (*n*) | 1462 | 1367 | | 1438 | | 1714 | |  |
| Model 1 | 1 | 0.99 (0.91, 1.08) | | 1.08 (0.99, 1.18) | | 1.40 (1.28, 1.52) | | < 0.001 |
| Model 2 | 1 | 0.99 (0.91, 1.08) | | 1.08 (0.99, 1.18) | | 1.35 (1.24, 1.48) | | < 0.001 |
| RRR-derived dietary pattern |  |  |  |  |  |  |  |  |
| Total (*n*) | 5827 | 5819 | | 5896 | | 5917 | |  |
| MetS (*n*) | 1255 | 1351 | | 1515 | | 1860 | |  |
| Model 1 | 1 | 1.10 (1.01, 1.21) | | 1.31 (1.20, 1.43) | | 1.76 (1.61, 1.92) | | < 0.001 |
| Model 2 | 1 | 1.09 (0.99, 1.19) | | 1.27 (1.16, 1.39) | | 1.67 (1.53, 1.82) | | < 0.001 |
| Participants with moderate to severe impaired kidney function (eGFR < 60 mL/min/1.73 m^2^) | | | | | | | |  |
| PCA-derived dietary pattern |  |  |  |  |  |  |  |  |
| Total (*n*) | 679 | 575 | | 471 | | 385 | |  |
| MetS (*n*) | 300 | 285 | | 227 | | 183 | |  |
| Model 1 | 1 | 1.28 (1.02, 1.60) | | 1.30 (1.02, 1.65) | | 1.31 (1.01, 1.70) | | 0.045 |
| Model 2 | 1 | 1.34 (1.06, 1.68) | | 1.32 (1.04, 1.69) | | 1.35 (1.04, 1.75) | | 0.037 |
| RRR-derived dietary pattern |  |  |  |  |  |  |  |  |
| Total (*n*) | 576 | 513 | | 572 | | 449 | |  |
| MetS (*n*) | 240 | 231 | | 278 | | 246 | |  |
| Model 1 | 1 | 1.17 (0.92, 1.49) | | 1.37 (1.09, 1.74) | | 1.85 (1.43, 238) | | < 0.001 |
| Model 2 | 1 | 1.23 (0.96, 1.57) | | 1.42 (1.12, 1.81) | | 1.85 (1.42, 2.39) | | < 0.001 |

PCA principal component analysis, RRR reduced rank regression, MetS metabolic syndrome, eGFR estimated glomerular filtration rate.

^a^ Data are presented as odds ratios (ORs) and 95% confidence intervals (95% CIs). Model 1: adjusted for age and gender. Model 2: adjusted for age, gender, education level, income, marital status, smoking, drinking, sleep quality, physical activity and cardiovascular disease status.
